# Detailed surgical description of porcine vascularized thymus lobe transplantation

**DOI:** 10.3389/frtra.2024.1499844

**Published:** 2024-11-22

**Authors:** M. Esad Gunes, Sho Fujiwara, Daniel H. Wolbrom, Alexander Cadelina, Susan Qudus, Dilrukshi Ekanayake-Alper, Dominik Hajosi, David H. Sachs, Greg Nowak

**Affiliations:** ^1^ Columbia Center of Translational Immunology, Columbia University, New York, NY, United States; ^2^ Department of Surgery, Columbia University, New York, NY, United States; ^3^ Department of Comparative Medicine, Yale University, New Haven, CT, United States; ^4^ Institute of Comparative Medicine, Columbia University, New York, NY, United States; ^5^ Department of Surgery, Massachusetts General Hospital, Boston, MA, United States; ^6^ Division of Transplantation Surgery, Karolinska Institute, Stockholm, Sweden

**Keywords:** swine, baboon, thymus transplantation, vascularized thymic lobe, allotransplantation, xenotransplantation, tolerance

## Abstract

**Background:**

Despite advances in immunosuppressive therapies, chronic rejection and immunosuppression-related complications remain significant challenges in transplantation. Developing transplantation tolerance through thymus transplantation may offer a solution. This paper details our technique for procuring and transplanting porcine vascularized thymic lobes (VTL), which can be utilized to study and research allogeneic and xenogeneic transplantation models in large animals.

**Methods:**

GalT-KO miniature swine (*n* = 16) and baboons (*n* = 12) were used for VTL transplantation. The right or left cervical thymic lobe was dissected, harvested with its artery and veins, and flushed with cold lactated Ringer's solution. VTL graft was transplanted intraabdominally in all animals.

**Results:**

We performed non-survival (*n* = 2) and survival (*n* = 2) VTL autotransplants in pigs and xeno-VTL and kidney transplants in baboons (*n* = 12). All grafts immediately turned pink after reperfusion and had good blood inflow and outflow. Pigs in the survival autotransplant group were euthanized immediately post-operatively due to complications related to VTL donation. One baboon lost its graft due to antibody-mediated rejection, and another lost it due to venous thrombosis. Other baboons had no complications and survived until the endpoint.

**Conclusion:**

Here, we describe our approach and experience in swine vascularized thymic lobe procurement and transplantation. The technique requires moderate surgical skills to achieve reproducible results. Living-donor VTL donation in pigs is not recommended due to the high risk of surgical complications related to the harvesting procedure.

## Introduction

Since the first successful solid organ transplantation series in the 1950s (1), transplantation has transitioned from a groundbreaking medical procedure to a widespread standard procedure, thanks to improvements in surgical techniques, immunology, and modern antibiotics and immunosuppressive drugs (2). Even though graft survival in solid organ transplantation has continuously improved, its long-term survival is limited due to the occurrence of chronic rejection and immunosuppression-related complications. One potential solution may be the development of transplantation tolerance.

Transplant tolerance is defined as graft acceptance and stable function without immunosuppression in the presence of an otherwise functioning immune system (3). It has previously been shown that donor-specific tolerance can be achieved through mixed chimerism and thymus transplantation (TTx) under certain immunosuppression regimens (4–6). Of these methods, TTx has received considerable attention in developing operational tolerance in large animal models of allo- and xenotransplantation (7–10). Two main approaches have been taken to investigate thymus transplantation for the development of central tolerance in large animals across allo- and xenotransplantation barriers: (1) transplanting a porcine thymus as part of a composite graft, in which thymic fragments are implanted under the kidney capsule, where they develop vasculature and form the so-called thymokidney (11), (2) transplanting a porcine vascularized thymic lobe (VTL) (5).

In 2002, LaMattina et al. introduced VTL transplantation in a pig-to-pig model to investigate the development of tolerance through thymus transplantation in large animals (5). It is important to stress that, from the beginning, VTL transplantation from pig donors included transplantation of one of the cervical thymic lobes, a distinguishing difference in swine thymus anatomy compared to other large animals used in experimental transplantation. In 2004, Kamano et al. and Yamamoto et al. extended this procedure to a pig-to-baboon model to investigate tolerance development to xenografts (6, 12). Johnston et al. also described a related procedure that involved the transplantation of an *en-bloc* heart and vascularized thymus; however, this procedure did not include the isolation of a VTL as a graft (13). Recently, Yamada et al. described the VTL transplantation procedure concomitant with kidney xenotransplantation in 2020 (7). In this paper, we summarize our experience with VTL transplantation and describe in detail the surgical approach to VTL procurement and transplantation in pig-to-pig and pig-to-baboon models useful for investigating tolerance induction via thymic transplantation in large animals across allo- and xenograft barriers.

## Methods

### Animals

We utilized GalT-KO Sachs miniature swine (*n* = 16) weighing 16–30 kg as VTL donors and recipients (*n* = 4). Their properties have previously been reported (14, 15). Baboons (*Papio anubis* and *Papio hamadryas*) (*n* = 12) weighing 5–16 kg with varying degrees of natural non-Gal anti-swine antibodies were used as primate VTL recipients. VTL transplantations were followed by kidney transplantation from the same donor during the same surgical procedure. All animal use has been approved by the Institutional Animal Care and Use Committee (IACUC) at Columbia University under the IACUC protocol numbers AC-AABN1550 and AC-AABI7554.

### Experimental groups

Animals were divided into three experimental groups. Group 1 (*n* = 2) included two non-survival auto-VTL transplants to validate our technique. Group 2 (*n* = 2) included two survival auto-VTL transplants to investigate whether VTL donations could be performed as living donor donations. Group 3 (*n* = 12) included survival pig-to-baboon VTL xenotransplants.

### VTL dissection and procurement

The swine's chest and neck vascular anatomy allows for the dissection and harvest of both cervical thymic lobes as vascularized grafts without requiring a sternotomy. Since the dissection and harvest of both the left and right lobes are very similar surgically and technically, we herein describe a right VTL harvest only.

**Figure 1:**
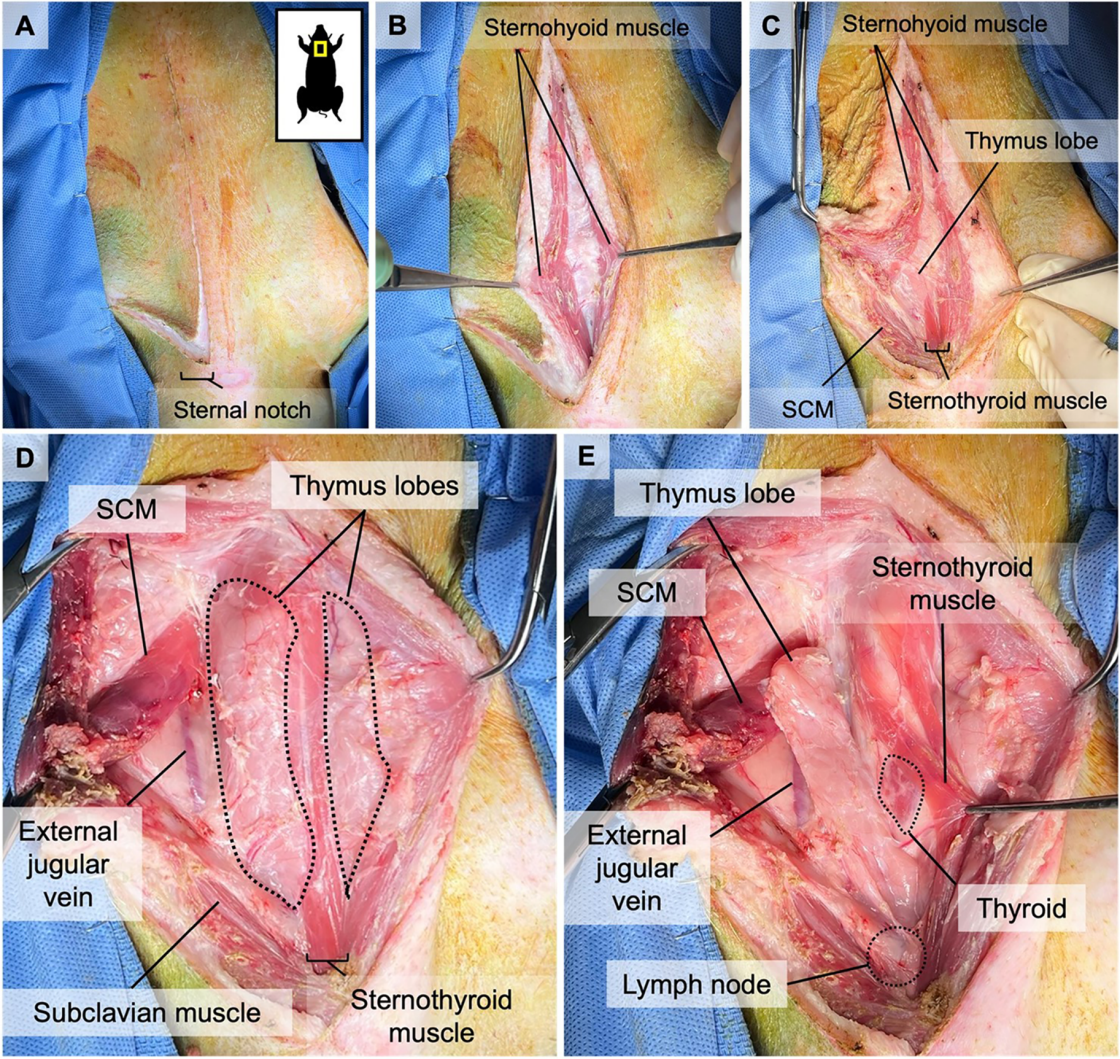
Surgical exposure of a right VTL graft.

**Figure 2:**
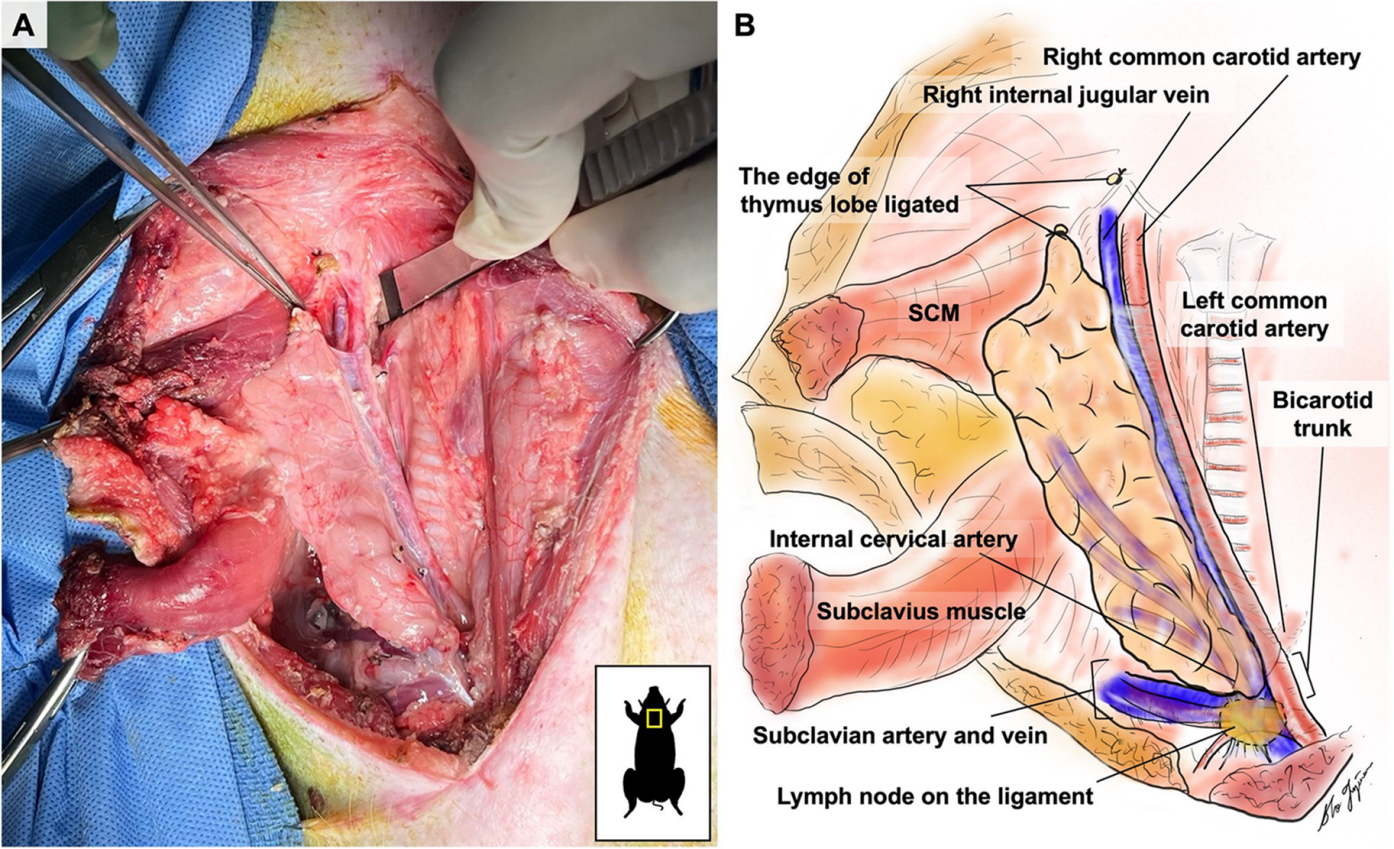
Medial border view of a right VTL graft after dissection.

**Figure 3:**
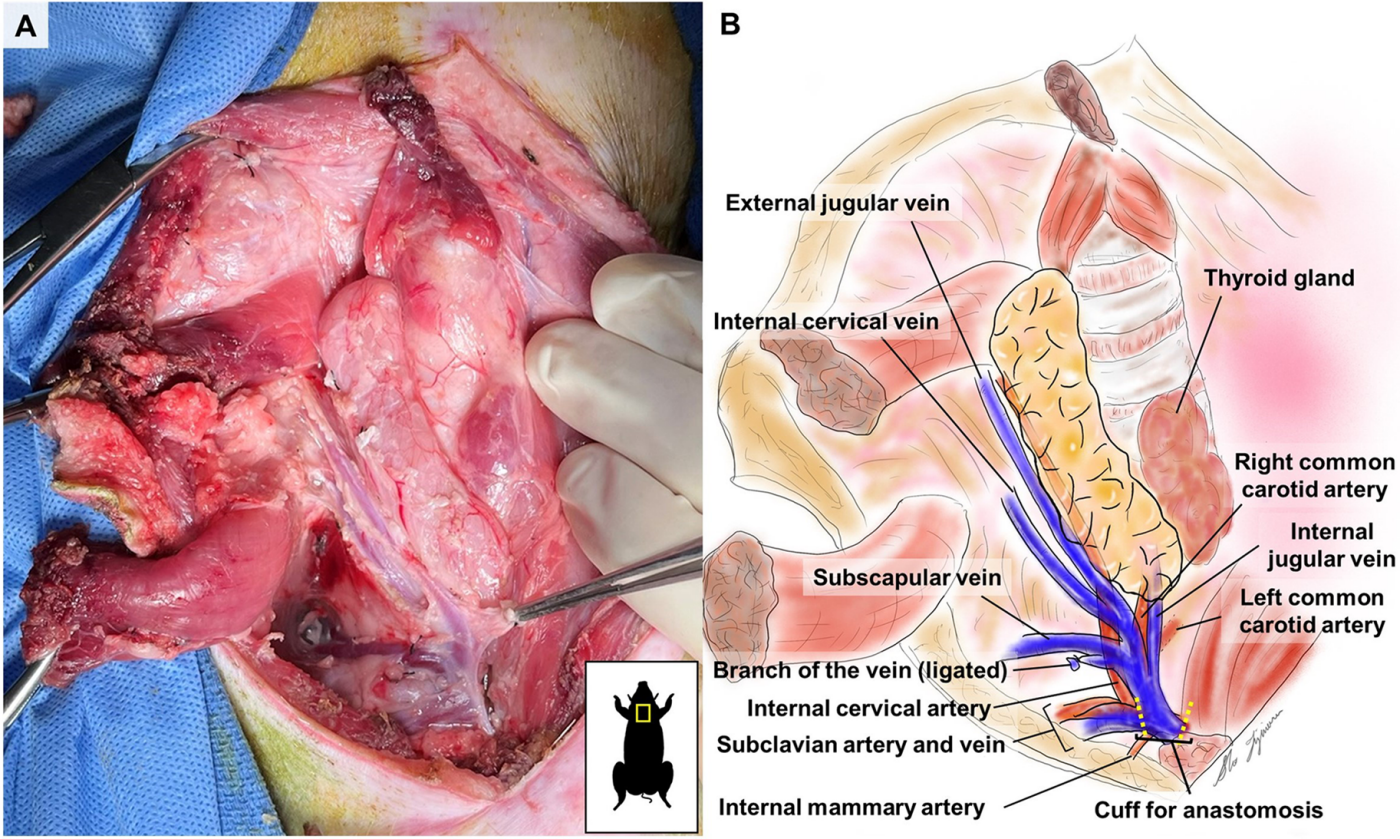
Lateral border view of a right VTL graft after dissection.

The animal is sedated and intubated, and its neck is draped in a triangle, extending from both humeral heads to the superior aspect of the larynx. 100 U/kg of heparin is administered IV before the start of the surgery to prevent the development of any microthrombi in the graft during the dissection. Afterward, a reversed L-shaped incision is made from the superior edge of the larynx down to the sternal notch in the middle line, with extension to the right humeral head parallel to the upper edge of the right clavicle (Figure 1A). Dissection continues until the sternohyoid muscles are identified. The connective tissue between the sternohyoid muscles is dissected to reveal the thymic lobes (Figure 1B). The left sternohyoid muscle is retracted leftwards to increase visibility. The right sternohyoid and sternocleidomastoid muscles are stripped from their sternal insertions and gently dissected off the right thymic lobe to visualize that lobe (Figures 1C,D). Thereafter, the right sternothyroid muscle is gently dissected from the thymus to allow access to the carotid sheath (Figures 1D,E). The subclavius, omohyoid, and superficial pectoral muscles are bluntly transected close to their insertion points between the sternal notch and the humeral head to allow better access to subclavian vessels (Figures 2, 3).

On the medial side, the thymic lobe is dissected off the trachea and thyroid until the carotid sheath is identified (Figure 2). The common carotid artery is dissected off the thymic lobe and internal jugular vein (IJV) as it does not supply any branches to the thymic lobes and might cause stasis and thrombosis if transplanted with the VTL graft. On the lateral side, dissection continues until the external jugular vein (EJV) is identified. Once the EJV is identified, the dissection continues on the lateral side of the EJV, extending laterally to the internal cervical vein (ICeV) to ensure that the draining microvessels of the thymus are not disrupted (Figure 3). The right subscapular vein is ligated distal to the confluence of EJV and ICeV. The right cervical thymus lobe is ligated and divided distally at the level of the waist between the distal and main body parts of the cervical thymic lobe, as the superior cervical thymic lobe receives its arterial blood supply elsewhere (Figure 2). The connection between the right and left thymus lobe or intrathoracic thymus is ligated and divided. The prevertebral attachments of the thymic lobe (posterior wall of the thymic lobe) are dissected and ligated to complete the dissection of a thymic graft *en-bloc* with IJV, EJV, and ICeV constituting its venous drainage.

Attention is shifted to the dissection of the subclavian artery (SA) and the subclavian vein (SV), positioned just behind the supraclavicular lymph nodes, which can be used as a landmark (Figure 2). The supraclavicular lymph nodes are excised to visualize the SA and SV better. Gentle dissection is performed to free the confluent portion of the SV draining IJV, EJV, and ICeV, which constitutes the venous outflow of the VTL grafts (Figures 2, 3). The SA, situated behind the SV, is gently dissected to isolate the internal thoracic artery and to identify the right internal cervical artery (ICeA), which constitutes the arterial inflow to the main body of VTL right lobe thymus grafts (Figures 2, 3).

When the graft is ready to be explanted, 400 U/kg of heparin is administered intravenously. Subsequently, the distal IJV, EJV, and ICeV are divided between ligatures at the level of the superior pole of the thymic lobe (Figures 2, 3). The internal thoracic vein and arteries are divided distally between ligatures (Figures 2, 3). The subclavian vein is either clamped or ligated proximally to the IJV and transected (Figures 2, 3). Continuous bleeding from the venous patch confirms that the graft has good arterial circulation. The subclavian artery is either clamped or ligated in both distal and proximal sections to the ICeA and transected with a patch (Figures 2, 3). The VTL graft is then removed, placed on ice, and flushed with cold Lactated Ringer's Solution through the ICeA.

### VTL transplantation

**Figure 4:**
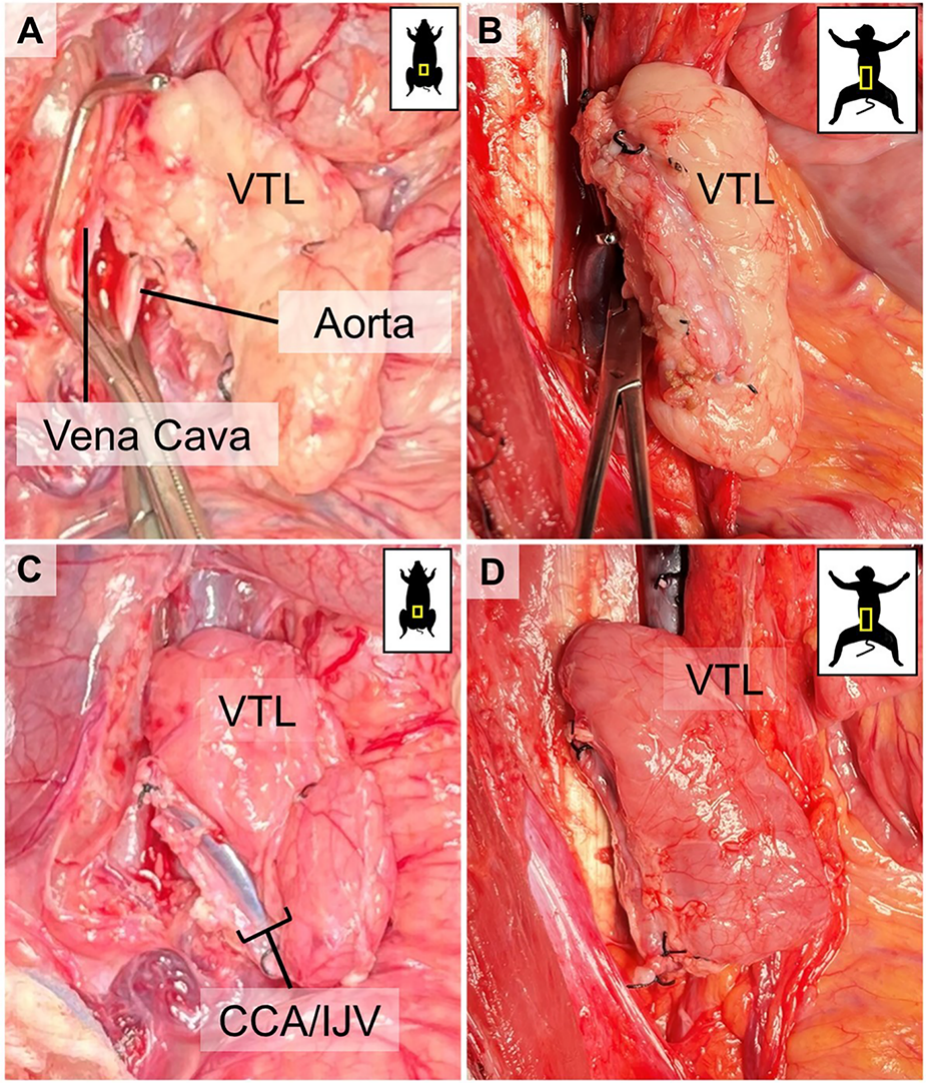
Visual representation of VTL grafts before and after reperfusion. **(A)** corresponds to **(C)** before unclamping and reperfusion in a pig-to-pig, and **(B)** corresponds to **(D)** before unclamping and reperfusion in a pig-to-baboon VTL transplantation. Notice the immediate change in the color of the graft, indicating successful reperfusion.

The recipient animal is sedated, intubated, and draped. 100 U/kg of heparin is administered IV before the start of the surgery, and a repeated dose is administered during VTL implantation. A midline abdominal incision is made. Dissection continues until the abdominal cavity is entered. The bowel is retracted to the left, and the infrarenal aorta and vena cava are dissected and prepared for anastomosis. Satinsky clamps are used to side-clamp the vena cava and aorta. The venous anastomosis is performed using 7/0 Prolene as a continuous suture. The ICeA, constituting the inflow of the VTL graft, is anastomosed on a patch using 7/0 Prolene as a continuous suture. In 1 animal in Group 2, the ICeA was anastomosed using interrupted 7/0 Prolene sutures. Venous and arterial clamps are removed to allow reperfusion of the graft, respectively (Figure 4).

## Results

### VTL transplantation in swine (groups 1 and 2)

We initially performed two non-survival auto-VTL transplants (Group 1) to prove and validate our approach and technique. The VTL graft was harvested and transplanted on the infrarenal aorta and vena cava of the same pig, as described above (Figures 4A,C). After the removal of the cross-clamp, ICeA on the graft side was pulsatile, indicating good inflow, and the graft remained pink, indicating good outflow and no congestion (Figures 4A,C).

Furthermore, we investigated whether VTL donations in a living-related fashion could be performed to allow for delayed organ transplantation from the same donor following tolerance induction by transplanted VTL. We performed two auto-VTL transplants (Group 2) as described above, with additional reconstruction of the subclavian artery and vein. Animal 1 received a VTL with a patch, requiring a resection of a portion of the subclavian artery. Animal 2 received a VTL without a patch, which spared the subclavian artery but required interrupted sutures for arterial anastomosis in the recipient. In both animals, the VTL graft turned pink immediately and appeared to have good perfusion. Following the surgery, Animal 1 was able to recover from surgery and anesthesia, while Animal 2 was not able to recover from anesthesia, probably due to acute intracranial edema, and the animal was euthanized under the same anesthesia. Animal 1, immediately after the surgery, lost the function of its extensor muscles in its front legs, positioning in hyperflexion, likely due to damage to the brachial plexus. Therefore, the animal was euthanized shortly after recovery from the anesthesia.

### VTL transplantation in primates (groups 3)

Once we successfully established VTL graft procurement with good VTL reperfusion in the allotransplantation model, we performed pig-to-baboon VTL transplants (Group 3, *n* = 12). We utilized GalT-KO pigs as VTL donors and either *Papio anubis* or *Papio hamadryas* baboons as recipients. The VTL grafts were harvested from the donors and transplanted infrarenally to the baboon recipients, as described above. After the removal of the cross-clamp, all grafts were well-perfused without any evidence of congestion (Figures 4C,D). One graft underwent acute antibody-mediated rejection immediately after transplantation, while another graft suffered acute venous thrombosis after the transplant, requiring VTL explantation on postoperative day 6, which was well tolerated by the animal with full recovery after that. In 3 of the earlier transplanted animals, at the time of euthanasia, we found that the venous drainage was occluded entirely due to what we believe to be chronic venous stasis and thrombosis. The course of venous complications, surgical and, or immunological, is still under investigation. All VTL transplants in baboons were followed by kidney transplantation from the same donor, the results of which will be published elsewhere.

## Discussion

We described here our approach to VTL harvesting and transplantation from swine in detail. We also investigated the possibility of performing VTL donations in a living-related fashion with donor survival post-surgery.

Using our surgical technique, we successfully procured and transplanted VTL grafts, all of which were well-perfused immediately after the cross-clamp was removed. Furthermore, we found that after transplantation, especially in the primates, VTL vein thrombosis seemed to be a common surgical complication (5, 6, 12). Although this complication is one of the leading surgical complications in microsurgery and transplantation, we cannot exclude the involvement of xenotransplantation-specific immunoreaction that increases the risk for such complications in xenotransplantation. Furthermore, we hypothesized that venous thrombosis in VTL xenograft can be also related to physiological and anatomical differences between pigs and baboons. The cervical thymic lobes in a pig are positioned horizontally immediately above the heart, where the cervical thymic lobes have a net negative pressure in its draining vessels, which prevents stasis of the blood. This is evident by the “pulsative” vein motion due to negative pressure visible in Supplementary Video S1. In contrast, the VTL graft is transplanted into the abdomen of a baboon, which, unlike a pig that stands on four legs, sits upright and therefore VTL graft is now positioned vertically with constant positive pressure in its draining vessel, leading to a functional stasis of blood in VTL and likely increases the risk for venous thrombotic complications. A way to address this problem could be the transplantation of the VTL graft into the neck of a baboon, as it was previously done in pigs (6). However, since baboons used in transplantation research are very small (4–10 kg), there is not enough space for the VTL graft to be transplanted without compression from the surrounding tissue. Accordingly, to prevent thrombosis events in transplanted VTL grafts, we started continuous heparin infusion at a dose of 300 U/kg/day heparin for 30 days after transplantation. No complications due to the use of heparin were observed, and no thrombosis was diagnosed in animals that received heparin treatment.

We also investigated living-donor VTL donation as a potential alternative for thymus-induced tolerance protocols with delayed organ transplantation (e.g., skin, kidney) from the same donor. As such, it would have been valuable to harvest VTL and keep the donor animal alive until delayed organ donation and transplantation to the same recipient with induced tolerance by previously transplanted VTL from the same donor. However, due to the localization of the VTL graft and its relation to nerves and vasculature, VTL harvesting without damage to vital vessels and nerves, such as the brachial plexus and subclavian artery and vein, remains challenging even with reasonable experience in microsurgery and VTL procurement.

To summarize, we have described our technique for swine VTL harvest and transplantation in allo- and xenotransplantation models and believe that our detailed description will help guide other investigators in their research efforts to establish VTL transplantation models and investigate further thymus-mediated tolerance. Our VTL xenotransplantation model also included kidney xenotransplantation, and the results of these experiments will be described elsewhere.

## Data Availability

The original contributions presented in the study are included in the article/Supplementary Material, further inquiries can be directed to the corresponding author.
